# Do Not Forget Poly (Adenosine Diphosphate-Ribose) Polymerase Inhibitors in Ovarian Carcinosarcoma

**DOI:** 10.7759/cureus.26662

**Published:** 2022-07-08

**Authors:** Denise Magalhães, Carla Bartosch, Miguel H Abreu

**Affiliations:** 1 Oncology, Hospital Pedro Hispano, Matosinhos, PRT; 2 Pathology, Instituto Português de Oncologia Francisco Gentil Porto, Porto, PRT; 3 Oncology, Instituto Português de Oncologia Francisco Gentil Porto, Porto, PRT

**Keywords:** platinum-sensitive disease, brca mutation, parp inhibitor, carcinosarcoma, ovarian cancer

## Abstract

Ovarian carcinosarcoma (OCS) is a rare entity with a poor prognosis and without evidence-based therapy. Here, we report the case of a 55-year-old woman with a germline *BRCA1* mutation and a stage IV OCS who was proposed olaparib maintenance therapy after three platinum-based chemotherapies in relapsed disease. Currently, the patient has an overall survival of 102 months and progression-free survival of 60 months with olaparib, with a good quality of life and not experiencing any adverse events. Despite the lack of evidence for the use of poly (adenosine diphosphate-ribose) polymerase inhibitors in OCS, our case report proves that patients with a potential biomarker of response to these drugs (such as *BRCA* mutation and platinum-sensitive disease) derive great benefits from it.

## Introduction

Ovarian cancer is the third most common and the second most lethal cancer of the gynecologic tract [1]. Ovarian carcinosarcoma (OCS), also known as mixed malignant Mullerian tumor, is characterized by the presence of both an epithelial and a mesenchymal component. This rare tumor accounts for 1-4% of all ovarian cancers. It is most frequently diagnosed in postmenopausal women aged between 60 and 70 years at an advanced stage [2,3]. At presentation, abdominal and/or pelvic pain, bloating, abdominal distention, and gastrointestinal symptoms are the most reported [3]. OCS are tumors with a poor prognosis, with a five-year overall survival (OS) of 28% [4].

Given the rarity of OCS, it has been challenging to develop recommendations for its management. Prospective trials are lacking, with most knowledge derived from limited retrospective data and extrapolation from epithelial ovarian cancer (EOC) experience [2,3,5]. Thus, international guidelines recommend treating OCS as an aggressive EOC [6].

Poly (adenosine diphosphate-ribose) polymerase (PARP) inhibitors are well established in EOC as maintenance therapy following response to platinum-based chemotherapy. Unfortunately, there are no data for PARP inhibitor therapy in OCS [6,7].

We aim to expand the knowledge regarding the management of OCS by presenting a case with sustained response to maintenance therapy with PARP inhibitors.

## Case presentation

Our patient was a 55-year-old woman without any relevant personal history. Concerning familial history, the patient’s sister had bilateral breast cancer diagnosed at 52 years of age. In the context of abdominal pain, a large pelvic lesion (21 × 10 cm) was noted near the right ovary, associated with cancer antigen-125 (CA-125) elevation (1,094 U/mL; normal: <35 U/mL). In March 2013, the patient underwent an exploratory laparotomy. A large tumor mass, with involvement of the Fallopian tube and extension to the small intestine and abdominal wall, was identified. A right salpingectomy, segmental enterectomy, and omentectomy were performed; however, diffuse tumor disease remained.

**Figure 1:**
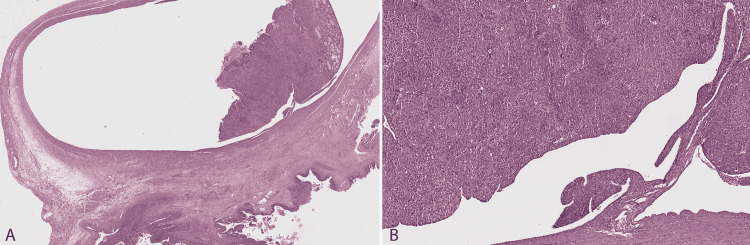
Histological features of carcinosarcoma in the salpingectomy specimen. (A) The tumor is present within the Fallopian tube lumen with a polypoid appearance and involves its serosa. (B) Higher amplification showing the point of continuity between the remaining Fallopian tube mucosa and the tumor, mostly consisting of the epithelial component (high-grade endometrioid carcinoma) with a solid pattern.

**Figure 2:**
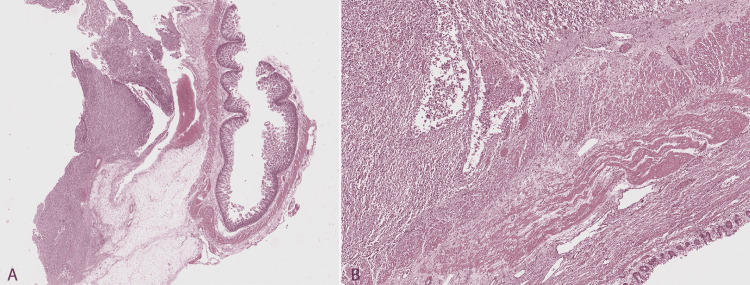
Histological features of carcinosarcoma in the enterectomy specimen. (A) Tumor implant in the mesenteric adipose tissue. (B) Tumor involving the enteric serosa and focally invading the outer muscle layer of the intestinal wall. Here the tumor consists predominantly of the mesenchymal component (undifferentiated sarcoma).

**Figure 3:**
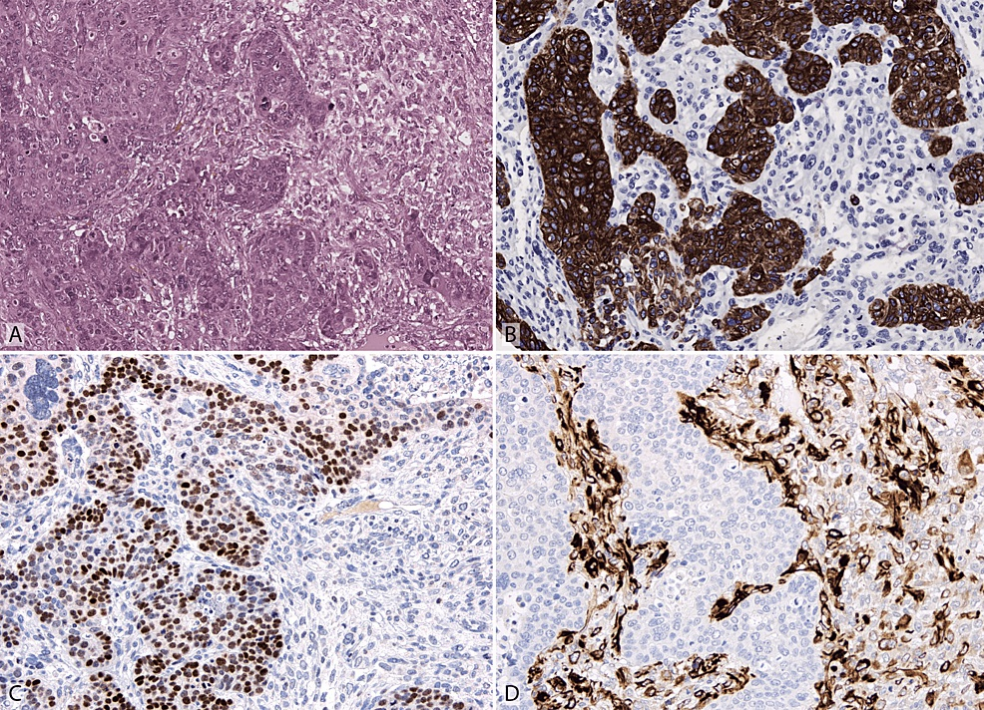
Histological and immunohistochemical features of carcinosarcoma. (A) The tumor consisted of two intermixed components with features suggestive of high-grade carcinoma endometrioid (on the left) and undifferentiated sarcoma (on the right). (B) Strong expression of cytokeratin 8/18 in the carcinoma negative in the sarcoma. Several mitoses are evident in the sarcoma component. (C) Patchy expression of estrogen receptor in the carcinoma areas. (D) Negative Wilms tumor 1 in the carcinoma, with cytoplasmic expression in the sarcoma.

Pathological examination showed a carcinosarcoma at the Fallopian tube (Figure 1) involving the Fallopian tube serosa, enteric serosa, omentum, and mesenteric adipose tissue. Focally, the tumor invaded the outer muscle layer of the enteric wall (Figure 2). It was composed of an epithelial and a mesenchymal component that constituted around 60% and 40% of the tumor, respectively. The epithelial component had features suggestive of high-grade endometrioid carcinoma, showing diffuse CK8/18, abnormal (negative) p53, patchy estrogen receptor (ER) staining, and negative Wilms tumor 1 (WT1) (Figure 3). The mesenchymal component had features suggestive of undifferentiated uterine sarcoma, showing abnormal (negative) p53, WT1 cytoplasmic expression and absence of CK8/8, ER, desmin, myogenin, and MyoD1.

Thoracoabdominal-pelvic computed tomography (TAP-CT) revealed a high residual tumor burden showing one liver lesion (2.9 cm) with capsular involvement, a retroperitoneal lesion (5.7 × 4.6 cm), a left iliac lesion (6.1 × 6 cm)\, and peritoneal implants. The patient was admitted to our institution in May 2013. Overall, the patient was staged as International Federation of Gynaecology and Obstetrics (FIGO) IVB. The disease was considered unresectable and the patient was proposed first-line palliative chemotherapy with carboplatin (area under the curve (AUC) = 6) and paclitaxel (175 mg/m_2_) every three weeks. After eight cycles, in October 2013, the patient achieved the best response as partial response (residual left iliac lesion) and presented CA-125 normalization (29.9 U/mL) without relevant toxicity. The patient started surveillance.

Nine months after the last chemotherapy cycle, the disease progressed to a left iliac lesion and pre-cava adenopathy. The patient started second-line chemotherapy with the same carboplatin-based doublet. The patient completed eight cycles in January 2015 achieving a partial response and remained in surveillance.

After 11 months of surveillance, the patient had a similar progression in the left iliac lesion and pre-cava adenopathy, and elevation in CA-125 occurred (23 U/L to 150 U/L). Third-line chemotherapy was started in January 2016, with the previous scheme. After six cycles, fluorodeoxyglucose (FDG)-positron emission tomography (PET)/CT scan showed a clear decrease in the iliac lesion and retroperitoneal adenopathy metabolism.

Genetic testing revealed a germline *BRCA1 *mutation. It was then considered that the patient could benefit from PARP inhibitor therapy, so it was decided to start olaparib 400 mg twice daily in September 2016.

**Figure 4:**
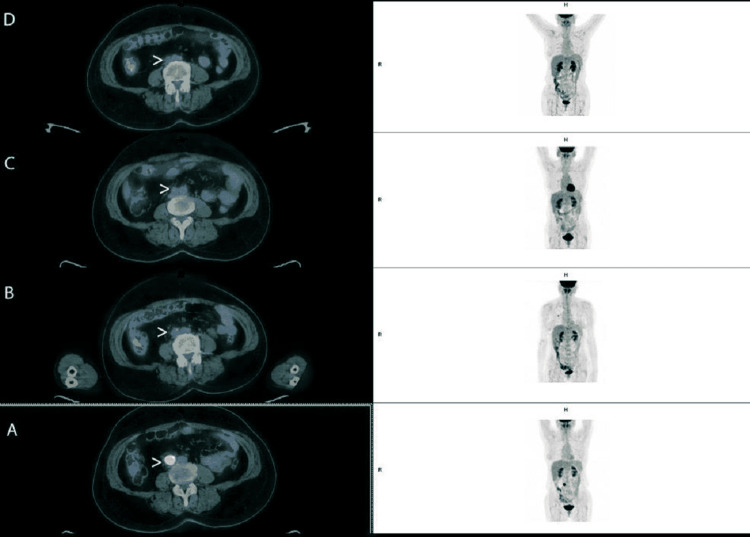
Retroperitoneal adenopathy evolution on fluorodeoxyglucose-positron emission tomography scan. Arrow points to the retroperitoneal adenopathy. (A) image before third-line platinum-based chemotherapy. (B) Response evaluation after six cycles of third-line chemotherapy showing a reduced metabolic activity at retroperitoneal adenopathy. (C) Response evaluation after six months of olaparib documenting complete response. (D) Sustained complete response at 60 months of olaparib. R: right side; H: head

**Figure 5:**
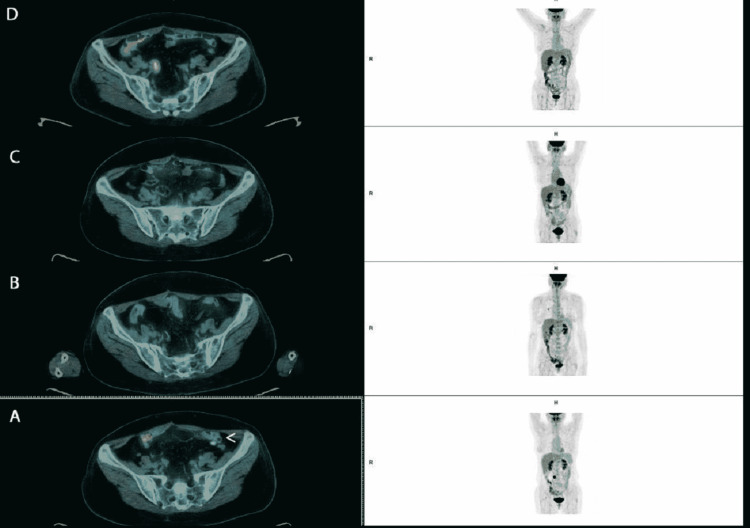
Evolution of mesenteric hypermetabolism on fluorodeoxyglucose-positron emission tomography scan images. Arrow shows mesenteric fluorodeoxyglucose hypermetabolism. (A) Before third-line platinum-based chemotherapy. (B) Response evaluation after six cycles of third-line chemotherapy showing complete response (no fluorodeoxyglucose uptake). (C) Sustained response at six months and (D) 60 months of olaparib. R: right side; H: head

Frist response evaluation performed in December 2016 showed an additional response after PARP inhibitor, achieving complete response (Figures 4, 5).

**Figure 6:**
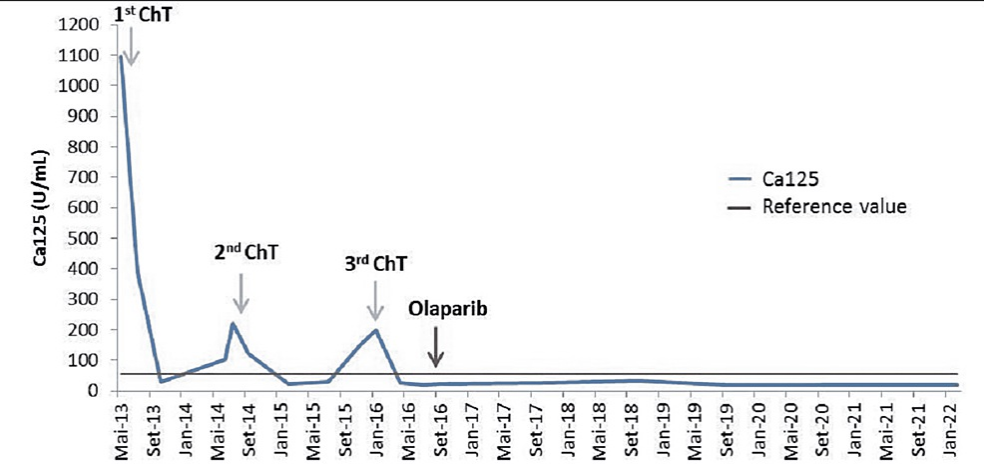
Evolution of serum CA-125 marker during cancer treatment. Arrow pointing to systemic treatment introduction. ChT: chemotherapy; CA-125: cancer antigen 125

In the last response evaluation, there was no evidence of progressive disease, and the tumor marker was consistently negative (Figure 6). Currently, the patient is asymptomatic without any adverse event related to olaparib (under full dose) and with an excellent quality of life. Presently, progression-free survival (PFS) (since the last chemotherapy line) is 64 months and OS is 106 months since first-line chemotherapy.

## Discussion

OCS are tumors with a poor prognosis when compared to EOC, with the median OS between 8 and 26 months and between 16 and 41 months, respectively [3,5]. Many prognostic factors have been suggested, including advanced age and stage, incomplete cytoreductive surgery, and sarcomatous component >25%. Additionally, the approach to these patients is similar to other ovarian carcinomas [2,5]. Our patient had an advanced-stage disease that precluded a cytoreductive surgery. In the advanced stage, cytoreductive surgery without residual disease, followed by chemotherapy, is the cornerstone of the treatment [2,5]. Regarding the best chemotherapy scheme, only a few retrospective trials have shown effectiveness in the platinum-taxane regimen, as well as the association of ifosfamide-taxane [2]. In these trials, patients treated with the platinum-taxane regimen presented a response rate ranging from 62% to 72% [2]. The GOG-0261 trial, a non-inferiority trial, compared carboplatin-paclitaxel with ifosfamide-paclitaxel. The trial showed similar OS but an improved PFS with carboplatin-paclitaxel treatment (15 months versus 10 months, respectively) [8].

Efforts are being made to improve OCS outcomes by exploring biomarkers that can provide improved outcomes and targeted therapy. Human epidermal growth factor receptor 2 (HER2) amplification was identified in 25-56%, epidermal growth factor receptor (EGFR) in 58%, and vascular endothelial growth factor (VEGF) in 44% of OCS cases [2,9]. *BRCA1/2* mutations are associated with an improved response to platinum-based chemotherapy (84% to 95% versus 60% in non-mutated patients), a persistent platinum sensibility, and a better OS (59 to 101 months versus 41 to 35 months in non-mutated patients) [9,10].

*BRCA1/2* mutations also predict response to PARP inhibitors in high-grade serous and endometrioid ovarian cancers. The exact rate of *BRCA1/2* mutations in OCS is not known. Pennington et al. demonstrated a loss-of-function mutation in homologous recombination genes (*BRCA1/2* and other *HDR*) in four of 12 (33%) OCS, a percentage similar to the other histological subtypes enrolled [9].

PARP inhibitors, as maintenance therapy, are now the gold standard for platinum-sensitive recurrent disease that presented with response to the last chemotherapy regimen. Four trials investigated the effectiveness of PARP inhibitors as maintenance therapy in recurrent disease; however, none of these had included OCS tumors, hence, evidence for its use in these patients is deficient [6,11]. A preclinical study demonstrated niraparib’s activity against OCS cell lines, harboring genetic signatures of *HRD*, but not for olaparib [12].

In the presented case, the patient had an extensive partial response to the frontline platinum-based chemotherapy and remained platinum-sensitive for three consecutive lines. This behavior leads to the investigation of *BRCA* mutation. Due to *BRCA1* germline mutation and platinum sensitivity, it was possible to administer olaparib as maintenance therapy (as off-label use). With PARP inhibitors, the patient had further tumor shrinkage. The antitumor activity of PARP inhibitors was studied with a reported objective response rate of 23% to 53% [13].

After 60 months, the patient continued to benefit from this treatment, with the disease under control and without any degradation in her quality of life. There was no need for dosage adjustment.

To our knowledge, this is the first case report of olaparib maintenance therapy in *BRCA*-mutated OCS, with an exceptional benefit, similar to the long responder already described in the clinical trials. There is another published case report of olaparib maintenance therapy in OCS, but in that case, the patient was *BRCA* non-mutated. The patient was administered olaparib for recurrent platinum-sensitive disease, with a progression after seven months of therapy [14]. By comparison, our patient had a better outcome, so the presence of biomarkers (*BRCA *mutation) could help to identify the population that would most benefit from this strategy. Olaparib constitutes a new approach in this population with few therapeutic options.

## Conclusions

This case reports an uncommon good prognosis in OCS, an aggressive tumor. It proves that even uncommon and untested by clinical trials ovarian cancer may derive great benefit from treatment with PARP inhibitors. Biomarkers such as *BRCA *mutation and platinum-sensitive disease could help to select the population that will benefit from this strategy.
